# Blended Trauma-Focused Cognitive Behavioral Therapy With Compassion for Adolescents With Posttraumatic Stress Disorder: Protocol for a Pilot Randomized Controlled Trial in Northern Sweden

**DOI:** 10.2196/92270

**Published:** 2026-07-15

**Authors:** Linda Wallin, Carl Göran Svedin, Inga Dennhag

**Affiliations:** 1 Child and Adolescent Psychiatry Section, Department of Clinical Science, Umeå University, Akutvägen 4C, Målpunkt ZC23, By 28, NUS, Umeå, 90187, Sweden, 46 0702101726; 2 Department of Social Work, Marie Cederschiöld University, Stockholm, Sweden

**Keywords:** PTSD, adolescents, blended therapy/intervention, trauma-focused cognitive behavioral therapy, compassion-focused therapy, telehealth/mHealth, pilot randomized controlled trials, posttraumatic stress disorder

## Abstract

**Background:**

Posttraumatic stress disorder (PTSD) affects up to 25% of trauma-exposed adolescents; yet, access to evidence-based treatment remains limited in rural regions. Trauma-focused cognitive behavioral therapy (TF-CBT) is the first-line intervention, but structural barriers such as long travel distances and therapist shortages hinder implementation. Digital and blended formats may improve accessibility, but evidence for adolescents with PTSD is limited. Shame and self-criticism are common following interpersonal trauma and can reduce engagement; compassion-focused strategies target these mechanisms and aim to enhance emotional safety.

**Objective:**

This protocol outlines a pilot randomized controlled trial (RCT) evaluating the feasibility of delivering blended trauma-focused cognitive behavioral therapy with compassion (bTF-CBT-C) for adolescents with PTSD in routine clinical care. A secondary aim is to assess the acceptability of the intervention among adolescents and caregivers. In addition, the study explores patterns and variability in clinical outcomes to inform the design of a future noninferiority trial.

**Methods:**

A 2-arm parallel-group pilot RCT will randomize 40 adolescents (12‐17 years) with *DSM-5* (*Diagnostic and Statistical Manual of Mental Disorders* [Fifth Edition]) PTSD to bTF-CBT-C or standard TF-CBT in routine child and adolescent psychiatric services. The intervention includes an approximately 5-week web-based stabilization phase, followed by 7‐14 therapist-led sessions delivered primarily via videoconference, with some in-person sessions. Primary outcomes will assess feasibility (recruitment, retention, adherence, data completeness, and adverse events) and acceptability (satisfaction, alliance, and qualitative interviews). Exploratory outcomes include PTSD symptoms, self-compassion, emotion regulation, depression, anxiety, suicidality, and dissociation. Assessments will be conducted at baseline, poststabilization, posttreatment, and 6-month follow-up. Feasibility and acceptability will be summarized descriptively. Exploratory analyses using analysis of covariance and mixed effects models will estimate variance parameters, confidence intervals, and descriptive change trajectories without hypothesis testing. Qualitative data will be analyzed using reflexive thematic analysis.

**Results:**

Recruitment started in February 2026, and data collection is projected to be completed by December 2028. Feasibility and acceptability outcomes, along with exploratory clinical patterns, will be reported in accordance with the CONSORT (Consolidated Standards of Reporting Trials) extension for pilot and feasibility trials.

**Conclusions:**

Findings will inform the refinement of the intervention, the assessment of trial feasibility, and the selection of outcomes for a fully powered noninferiority RCT. The study will also contribute to understanding how compassion-focused strategies may support emotional safety and engagement in trauma-focused treatment for adolescents.

## Introduction

Posttraumatic stress disorder (PTSD) is a common and impairing condition affecting up to 25% of trauma-exposed adolescents worldwide [1,2]. Access to evidence-based trauma-focused psychological interventions remains uneven, particularly in sparsely populated regions. In northern Sweden, long travel distances, waiting lists, and limited access to trained therapists restrict timely care [3,4]. These barriers make it difficult for adolescents to engage in regular in-person treatment.

Beyond these structural constraints, adolescents living in rural northern Sweden face additional contextual challenges that influence both help-seeking and engagement in treatment. In small communities, concerns about stigma, confidentiality, and being recognized when visiting health services can limit access to care [5,6]. At the same time, qualitative research indicates ambivalence toward fully digital interventions, as these formats reduce practical demands but may also be experienced as emotionally distant [5]. These findings underscore the need for care models that combine accessibility with relational and emotional support, such as blended interventions [5-8].

These structural barriers may not only limit access to care but also influence psychological processes relevant to treatment engagement. For example, limited or disrupted access to trauma-focused interventions may exacerbate feelings of shame, isolation, and self-criticism, which are common following interpersonal trauma [9] and may reduce engagement in trauma processing.

Trauma-focused cognitive behavioral therapy (TF-CBT) is the recommended first-line treatment for youth with PTSD and has demonstrated strong efficacy across diverse populations [10,11]. However, its conventional face-to-face delivery can be difficult to scale in sparsely populated regions. Digital and blended interventions have been proposed to address these challenges by increasing flexibility and reach, but evidence for adolescents with PTSD remains limited [12]. This gap is particularly evident for interventions that integrate therapist support and explicitly target key psychological mechanisms such as shame and self-criticism [9,13,14].

Despite the increasing use of digital mental health tools, few trauma-focused mobile applications have been developed for adolescents, and even fewer have been scientifically evaluated [12,15]. Existing applications are rarely integrated into structured treatment programs. This highlights the need for scalable tools that support stabilization and emotion regulation while complementing therapist-led care.

In addition to these technological gaps, important psychological mechanisms remain insufficiently addressed. Shame and threat-based emotions are common across PTSD presentations and are particularly pronounced following interpersonal trauma [9,16]. These processes may be especially salient during adolescence, a developmental period characterized by heightened social sensitivity and identity formation [17-19]. They can interfere with engagement in trauma processing [9,16]. Compassion-focused therapy (CFT) provides a mechanism-based framework for addressing these processes by reducing shame, strengthening emotion regulation, and promoting affiliative soothing [20,21]. Early findings in young people suggest that compassion-based approaches may enhance engagement in treatment [22-24]. However, such strategies have not yet been systematically integrated into TF-CBT for adolescents with PTSD.

To address these gaps, a blended trauma-focused cognitive behavioral therapy intervention with integrated compassion-focused components (bTF-CBT-C) was developed. This manualized intervention combines mobile health components, including web-based stabilization modules and therapist-guided video sessions, with selected in-person meetings. The design aims to reduce practical barriers to care while maintaining therapeutic alliance and supporting engagement in trauma-focused work. The approach is informed by adolescents’ preferences in rural northern Sweden, emphasizing flexibility, relational support, and emotionally attuned care [5].

The intervention builds on 3 previous studies. First, a qualitative study of rural youths’ experiences of trauma therapy [5] informed the design of the blended format, including the emphasis on flexibility, relational support, and emotional attunement. Second, validation of the Compassionate Engagement and Action Scale for Youth – Swedish Version (CEASY-SE) [25,26] guided the selection of compassion-related outcome measures. Third, a pilot randomized controlled trial (RCT) of digital CFT for young people [22,23] informed the development and integration of compassion-focused modules within the intervention. Together, these studies provide empirical and theoretical foundations for the current trial.

Given the structural barriers to accessing trauma-focused treatment in rural regions, the primary aim of the blended intervention is to improve accessibility and scalability rather than to increase clinical efficacy. A noninferiority design is therefore appropriate to evaluate whether comparable clinical outcomes can be achieved while reducing barriers to care [27]. If outcomes are comparable to those of standard TF-CBT while requiring fewer in-person visits and reducing the travel burden, the intervention may provide important service-level advantages.

Although integration of compassion-focused strategies may enhance outcomes, the evaluation focuses on whether effectiveness can be maintained while improving access. Should clinically meaningful improvements be observed, alternative trial designs, such as superiority trials, may be considered.

In this context, the current pilot study will inform the design of a future noninferiority trial by providing estimates of recruitment, retention, outcome variability, feasibility, and acceptability, thereby reducing uncertainty in key design parameters. These will also support the selection of primary outcomes, estimation of sample size requirements, and specification of an appropriate noninferiority margin.

The aim of this pilot RCT will be to evaluate the feasibility and acceptability of delivering bTF-CBT-C to adolescents with PTSD within routine child and adolescent psychiatric services.

The objectives will be (1) to assess the feasibility of delivering bTF-CBT-C in routine care (eg, recruitment, retention, and adherence); (2) to evaluate the acceptability of the intervention (eg, satisfaction ratings and qualitative feedback); and (3) to explore patterns of change in clinical and mechanism-related outcomes, including estimation of variability to inform the design of a fully powered noninferiority trial.

## Methods

### Study Design

This pilot RCT will use a 2-arm parallel-group design with a mixed methods approach. The primary purpose of the trial will be to evaluate the feasibility and acceptability of delivering bTF-CBT-C compared with standard TF-CBT. Consistent with recommendations for pilot and feasibility trials, the study will not be powered to detect clinical effectiveness. The trial has been registered at ClinicalTrials.gov (ID: 2024-07057-01MixadTFKBT).

The trial will follow the updated SPIRIT (Standard Protocol Items: Recommendations for Interventional Trials) 2025 guidelines (Checklist 1) [28] for trial protocols and the CONSORT (Consolidated Standards of Reporting Trials) extension for pilot and feasibility trials [29]. A total sample size of 40 participants was selected as appropriate for a pilot feasibility trial. Similar sample sizes have been used in prior pilot studies of trauma-focused and blended psychological interventions, supporting their suitability for estimating feasibility outcomes in this context [30,31]. These participants (adolescents aged 12‐17 years meeting criteria for PTSD) will be randomized to either bTF-CBT-C or standard TF-CBT in routine child and adolescent psychiatric services.

The study adopts a convergent mixed methods design, in which quantitative feasibility and acceptability outcomes are collected in parallel with qualitative interview data [32,33]. Integration will occur at the interpretation stage, where qualitative findings will be used to contextualize and explain quantitative patterns (eg, recruitment, adherence, and satisfaction) and to identify convergences and discrepancies across data sources.

Assessment time points and weekly process measures will follow the predefined SPIRIT schedule (Table 1), with additional operational details provided in Multimedia Appendix 1.

**Table 1.** SPIRIT^a^ schedule of enrollment, interventions, and assessments for the pilot randomized controlled trial bTF-CBT-C^b^ versus standard TF-CBT^c^.

Phase/activity	T0: baseline	T0a: allocation	T1: poststabilization	T2: postintervention	T3: 6-month follow-up
Enrollment					
Eligibility screening	✓				
Informed consent (youth + caregiver)	✓				
Baseline assessments	✓				
Randomization		✓			
Interventions					
bTF-CBT-C (experimental)		✓	✓	✓	✓
Standard TF-CBT (control)		✓	✓	✓	✓
Primary outcomes (feasibility)					
Recruitment rate	✓				
Retention rate			✓	✓	✓
Adherence (session attendance, module completion)			✓	✓	
Adverse events (youth, caregiver + therapist)			✓	✓	✓
Primary outcomes (acceptability)					
Satisfaction (youth + caregiver)				✓	
Therapeutic alliance (youth, caregiver, therapist)^d^			✓	✓	
Digital treatment evaluation (youth + caregiver)^d^			✓		
Exercise evaluation^d^			✓		
Qualitative interviews			✓	✓	✓
Exploratory and clinical outcomes					
PTSD^e^ symptoms (CATS-2^f^, CRIES-13^g^)	✓		✓	✓	✓
Dissociation (DSQ-12^h^)	✓			✓	✓
Self-compassion (CEASY-SE^i^)	✓		✓	✓	✓
Emotion regulation (DERS-16^j^)	✓			✓	✓
Depression/anxiety/suicidality (MADRS-Y^k^, RCADS^l^)	✓		✓	✓	✓
Progression criteria					
Recruitment ≥60%	✓				
Retention ≥70%			✓	✓	✓
Acceptability ≥4/5				✓	
Data completeness ≥80%			✓	✓	✓

a SPIRIT: Standard Protocol Items: Recommendations for Interventional Trials.

b bTF-CBT-C: blended trauma-focused cognitive behavioral therapy with compassion.

c TF-CBT: trauma-focused cognitive behavioral therapy.

d Repeated mechanism/process measures (eg, therapeutic alliance, digital treatment evaluation, exercise evaluation, shame visual analogue scale, Trauma-Related Shame Inventory/Trauma-Related Guilt Inventory) are collected throughout the stabilization and trauma phases. A detailed full schedule table with all weekly/mid-module assessments (T0-T9) is provided in Multimedia Appendix 1.

e PTSD: posttraumatic stress disorder.

f CATS-2: Child and Adolescent Trauma Screen, version 2.

g CRIES-13: Children’s Revised Impact of Event Scale, 13-item version.

h DSQ-12: Dissociative Symptoms Questionnaire, 12-item version.

i CEASY-SE: Compassionate Engagement and Action Scale for Youth – Swedish Version.

j DERS-16: Difficulties in Emotion Regulation Scale, 16-item version.

k MADRS-Y: Montgomery-Åsberg Depression Rating Scale for Youth.

l RCADS: Revised Child Anxiety and Depression Scale.

### Setting

The study will be conducted in child and adolescent psychiatry clinics in the regions of Norrbotten and Västerbotten in northern Sweden. These clinics provide publicly funded specialist mental health care for children and adolescents, including psychological and psychiatric assessment and treatment. All study procedures, including screening, recruitment, assessments, and intervention delivery, will be carried out within existing clinical infrastructure and coordinated by trained clinical staff at the participating units.

### Participants

#### Inclusion Criteria

Participants will be included if they meet the following criteria: (1) adolescents aged 12‐17 years; (2) *DSM-5* (*Diagnostic and Statistical Manual of Mental Disorders* [Fifth Edition]) PTSD diagnosis confirmed by the Mini-International Neuropsychiatric Interview for Children and Adolescents (MINI-KID) [7]; (3) score ≥25 on the Child and Adolescent Trauma Screen, version 2 (CATS-2) [8]; (4) a nonoffending caregiver willing to participate; and (5) ability to communicate in Swedish.

#### Exclusion Criteria

Participants will be excluded if they meet any of the following criteria:

1. Safety-related criteria: active psychosis, severe dissociative symptoms, and acute suicidality requiring inpatient care.
2. Comorbidity-related criteria: autism spectrum disorder, severe eating disorder, severe obsessive-compulsive disorder, substance use disorder requiring treatment, and regular benzodiazepine use (>1 time/week).
3. Logistical and treatment-related criteria: unstable living conditions or ongoing trauma exposure, cognitive impairments or medical conditions that prevent participation, concurrent trauma-focused psychotherapy, recent initiation or discontinuation of psychotropic medication (within 6 weeks), and planned medication changes during the study.

The exclusion criteria were selected to balance participant safety with the feasibility of delivering a blended trauma-focused intervention. Criteria related to acute suicidality, severe psychiatric comorbidity, and unstable living conditions were included to ensure that participants can safely engage in trauma-focused work. For example, recent changes in psychotropic medication and severe conditions such as eating disorders or obsessive-compulsive disorder were excluded to reduce clinical instability and potential confounding of treatment effects, particularly in a partially remote treatment format. Although these criteria may restrict eligibility in routine care, they were considered appropriate for this feasibility study in line with recommendations for pilot and feasibility studies [34]. Their impact on recruitment and external validity will be evaluated to inform future trials, including consideration of whether certain criteria may be modified or relaxed with appropriate monitoring strategies.

The study includes adolescents meeting *DSM-5* criteria for PTSD regardless of trauma type. Although compassion-focused mechanisms such as shame and self-criticism are particularly prominent in interpersonal trauma, these processes are also present across broader PTSD presentations. In specialist child and adolescent mental health services, where interpersonal and complex trauma exposures are common, these mechanisms are therefore relevant for a substantial proportion of participants. The intervention thus targets mechanisms that may be especially salient among adolescents exposed to interpersonal trauma while remaining theoretically and clinically relevant across broader PTSD presentations.

### Sample Size

As this is a pilot RCT, the sample size is set at 40 adolescents (20 per arm). This sample size is expected to enable estimation of key feasibility parameters, including recruitment, retention, adherence, and data completeness, as well as estimates of variability in outcome measures [34,35]. The selected sample size also reflects practical considerations related to recruitment within rural child and adolescent psychiatric services [36,37]. No formal power calculation will be conducted, as the trial is not designed to test intervention effectiveness but to inform the design and sample size planning for a future fully powered noninferiority trial.

### Ethical Considerations

The study will be conducted in accordance with the Declaration of Helsinki and applicable Swedish regulations governing research involving minors. Ethical approval has been obtained from the Swedish Ethical Review Authority (DNR 2024-07057-01, 2025-04566-02). Adolescents and caregivers will receive written and verbal information about study procedures, risks, benefits, confidentiality, and their right to withdraw from the study at any time without consequences to their clinical care. Written informed consent will be obtained from caregivers, and written assent or consent will be obtained from adolescents, depending on age and regional guidelines.

Data management will comply with the General Data Protection Regulation (GDPR) and institutional standards for handling sensitive personal data. All study data will be pseudonymized and stored on secure, access-restricted servers, with encrypted data transfer. Only authorized members of the research team will have access to identifiable data. Metadata and documentation will be prepared in accordance with the FAIR (findable, accessible, interoperable, and reusable) principles while ensuring that no identifiable information is publicly disclosed. Data will be retained in accordance with institutional requirements, and results will be disseminated through peer-reviewed publications and scientific presentations.

### Safety Procedures

A structured safety plan will be collaboratively developed with each adolescent and caregiver during the first therapist-led meeting, prior to the web-based stabilization phase. The plan will outline individualized strategies for managing distress, suicidal ideation, self-harm urges, and other safety concerns. It will include coping strategies, caregiver involvement, and clear instructions for contacting clinical staff during working hours or emergency services if risk escalates outside these hours.

During the web-based phase, suicide risk and distress will be monitored weekly via digital self-report measures completed by adolescents and caregivers. Reports indicating increased risk (eg, escalating distress, suicidal ideation, or self-harm urges) will trigger an alert to the clinical team. A clinician will review the alert within 1 working day, contact the adolescent and caregiver to assess risk, and initiate appropriate actions.

Therapists will follow a predefined protocol for managing interrupted or terminated video sessions. In the event of an unexpected disconnection, the therapist will first attempt to reconnect immediately, then contact the adolescent by telephone. If contact cannot be established, standard welfare-check procedures will be initiated, including contacting caregivers and, if necessary, coordinating with local services or emergency interventions.

Adverse events will be monitored through structured reports completed by adolescents, caregivers, and therapists, documenting occurrence and severity. All safety-related events, including suicide-related concerns, will be documented in a study safety log and reviewed in supervision. Serious adverse events (eg, suicide attempts, psychiatric hospitalizations, or significant clinical deterioration) will be reported to the principal investigator.

### Trial Status

Recruitment started in February 2026, with data collection expected to be completed by December 2028. This protocol reflects version 1.0, finalized in January 2026. At the time of submission (May 2026), 6 participants were enrolled.

### Digital Components

The bTF-CBT-C will use the CE-marked Amni web-based application [38] to deliver psychoeducation, digital exercises, and weekly self-report measures. Participants will access the application on their own smartphones or tablets. For example, adolescents and caregivers may engage with short films and guided audio-based exercises such as soothing rhythm breathing. They may also complete interactive self-compassion exercises that involve reflecting on self-critical thoughts or participate in brief gamified quizzes and tasks to reinforce learning and engagement. The application also includes ratings after exercises to capture engagement and perceived helpfulness. It collects symptom, process, and engagement data through secure, encrypted channels, providing real-time feedback to the clinical team. Screenshots from the application are provided in Multimedia Appendix 2.

Videoconferencing sessions will be conducted using GDPR-compliant platforms (Visiba Care and Platform24), integrated into routine clinical workflows. Technical support will be available through the clinic to assist participants with login issues, navigation, or other technical challenges related to digital components.

### Recruitment and Randomization

Participants will be recruited through child and adolescent psychiatry clinics in the regions of Norrbotten and Västerbotten. Clinicians or research staff will provide information about the study to potentially eligible adolescents and caregivers during routine clinical contacts, after which interested families will be invited to undergo eligibility screening and informed consent.

Following completion of baseline assessments, eligible participants will be randomized in a 1:1 ratio to either bTF-CBT-C or standard TF-CBT. An independent researcher not involved in recruitment or treatment will generate a computer-based randomization sequence using permuted blocks (block size=4) to maintain balance between study arms. Randomization will be stratified by geographic region to account for contextual differences between sites.

Allocation will be concealed using sequentially numbered, sealed, opaque envelopes prepared by the independent researcher. Envelopes will be opened only after baseline assessments are finalized. Due to the nature of psychological treatment, therapists and participants cannot be blinded to group assignment; however, outcome assessors will remain blinded whenever feasible to minimize assessment bias.

### Intervention Development and Framework

The development of the bTF-CBT-C intervention will follow the multiphase optimization strategy (MOST), a structured framework for developing and refining complex behavioral interventions [39,40]. MOST will be used to guide the preparation phase, including identifying core TF-CBT and compassion-focused components and contextual adaptations for adolescents in northern Sweden. The optimization phase will include iterative testing of web-based modules and therapist procedures, incorporating feedback from adolescents, caregivers, and clinicians to refine clarity, usability, and feasibility. The current pilot RCT will represent the evaluation phase, providing feasibility and acceptability data needed to finalize the intervention before a fully powered noninferiority trial.

### Cultural and Contextual Considerations

The intervention will be adapted to reflect cultural and contextual factors relevant to adolescents receiving mental health care in northern Sweden, including linguistic needs, local norms regarding help-seeking, and geographic considerations. Cultural tailoring will focus on ensuring accessibility, clarity, and relevance of both digital and therapist-led components. Therapist training will include a cultural competence module addressing minority and Indigenous perspectives, including Sámi cultural considerations. To support culturally responsive care, clinicians will incorporate brief questions inspired by the Cultural Formulation Interview to identify individual background factors that may influence engagement, communication, or treatment preferences. These adaptations are intended to strengthen relational safety and enhance feasibility and acceptability across diverse adolescent populations.

### User Involvement

User involvement will be integrated throughout the development and evaluation of the bTF-CBT-C intervention. Adolescents with lived experience of trauma, caregivers, and representatives from the Swedish user organization Hjärnkoll [41] will contribute to the design and refinement of the intervention and the trial procedures. Their input will inform the research plan, recruitment materials, application content, and the overall structure of the blended intervention program.

During the preparation phase, user representatives reviewed the preliminary research protocol, provided feedback on feasibility considerations specific to youth in northern Sweden, and participated in workshops to evaluate the clarity, accessibility, and acceptability of proposed modules. Their perspectives were incorporated into revisions of the intervention flow, digital components, and the caregiver involvement strategy.

User involvement will continue throughout the study, including consultation on implementation challenges, interpretation of qualitative findings, and dissemination of knowledge. This participatory approach aims to enhance ecological validity, cultural relevance, and acceptability across all phases of the project.

### Interventions

#### Overview

The two interventions share the same theoretical foundation in TF-CBT and its PRACTICE (psychoeducation, relaxation, affective modulation, cognitive coping, trauma narrative/exposure, in vivo mastery, conjoint caregiver-child sessions, and enhancing safety and future development) components [10,42] but differ in delivery format, structure, and focus on mechanisms.

Standard TF-CBT is delivered as therapist-led, clinic-based treatment, with sessions conducted in person at the clinic. bTF-CBT-C is delivered as a blended intervention that combines therapist-led sessions with planned digital components and the systematic integration of compassion-focused strategies.

#### Control Arm (Standard TF-CBT)

Participants randomized to the control arm will receive standard TF-CBT following the PRACTICE components [10,43]. Treatment will consist of approximately 12‐25 weekly therapist-led sessions delivered in person at the clinic. Only occasional individual sessions may be conducted via videoconferencing when required. Caregiver involvement will follow the procedures described in the TF-CBT manual.

Treatment fidelity in the control arm will be supported through adherence to the TF-CBT manual [10], regular supervision, structured session reports, and adherence checklists aligned with the PRACTICE components. These procedures mirror those used in the experimental arm to ensure comparable monitoring of treatment delivery.

#### Experimental Arm (bTF-CBT-C)

Participants randomized to the experimental arm will receive bTF-CBT-C. Treatment will consist of a blended intervention for adolescents and their caregivers that combines digital and therapist-led components, including therapist support during a web-based stabilization phase (via brief messaging and, if needed, stepwise escalation to telephone or videoconferencing) as well as approximately 7‐14 therapist-led sessions across the trauma processing and integration phases (primarily via videoconferencing with some in-person sessions). The intervention includes (1) a structured web-based stabilization phase, (2) systematic integration of digital modules throughout treatment, and (3) explicit incorporation of compassion-focused strategies targeting shame, self-criticism, and emotional safety. These mechanisms are expected to support engagement in trauma-focused work.

The intervention begins with an approximately 5-week stabilization phase delivered through structured web-based modules. Adolescents and caregivers have access to tailored application content relevant to their respective roles in treatment. This phase covers psychoeducation, relaxation, affective modulation, and cognitive coping and integrates compassion-focused strategies (eg, soothing rhythm breathing, compassionate imagery, and self-compassion exercises). Interactive exercises and nature-based mindfulness tasks aim to activate the soothing system and reduce threat-related responding. Gamified elements support engagement.

Following the stabilization phase, treatment continues with therapist-led trauma processing and integration components, including trauma narrative, cognitive restructuring, in vivo mastery, conjoint sessions, and safety planning, with caregiver involvement consistent with the TF-CBT model (C6-C9). Compassion-focused principles are applied throughout all phases of the intervention to support emotional safety and engagement.

While the intervention follows a predefined structure based on a manual, therapists can adapt pacing and delivery format (eg, videoconferencing or in-person sessions) based on clinical needs. Core treatment components are maintained and are considered mandatory for all participants. Deviations from the planned format (eg, missed sessions or reduced engagement) are systematically monitored and managed using a predefined, structured approach. To ensure consistency and transparency, adaptations are guided by predefined and clinically grounded principles, including symptom severity, participant engagement, and practical constraints such as geographic distance. Videoconferencing is used as the default format to enhance accessibility, particularly for participants in remote areas, whereas in-person sessions are prioritized when clinically indicated (eg, elevated risk, complex presentations, or reduced engagement). Missed or canceled sessions are systematically followed up, and the delivery format may be adjusted (eg, from videoconferencing to in-person sessions, or vice versa) to support continued engagement in treatment.

All adaptations to delivery format or pacing will be documented in structured treatment and session logs using predefined templates. Formal fidelity ratings are not included in this pilot study to minimize the burden on participants and therapists. Instead, fidelity will be supported through regular supervision, session reports, and adherence checklists aligned with the intervention manual. These checklists specify the delivery of core components within each treatment phase. Therapists will document the completion of core intervention components across phases, and adherence will be systematically reviewed in supervision to ensure that key elements are delivered as intended while allowing necessary adaptations.

A detailed description of modules, delivery format, and session content is provided in Table 2.

**Table 2.** Overview of bTF-CBT-C^a^ delivery across phases and modalities.

Phase/module	Delivery mode	Delivery details	Purpose of the module
Stabilization			
(C0) Introduction	Web-based application and clinic session	Self-paced onboarding; one-to-two 60‐90-minute clinic sessions with youth and caregiver	Establish therapeutic alliance, clarify structure, and initiate safety planning
(C1) Trauma and compassion	Web-based application and videoconference	Self-paced module; one 45‐60-minute videoconference session with youth and caregiver	Promote shared understanding of trauma and compassion; reduce shame through psychoeducation
(C2) Well-being and compassion with the body	Web-based application	Self-paced module; optional messaging support	Activate the soothing system and increase bodily awareness for emotion regulation
(C3) Emotions and the compassionate mind	Web-based application	Self-paced module; optional messaging support	Strengthening emotional literacy and tolerance through compassionate framing
(C4) Compassionate thinking	Web-based application	Self-paced module for youth and caregiver/messages; optional messaging support	Support cognitive coping and reduce self-criticism via compassionate self-talk
(C5) Preparing for trauma narrative with compassion	Web-based application	Self-paced module; optional messaging support	Built emotional scaffolding and readiness for trauma work
Trauma processing			
(C6) Trauma narrative with compassionate	Videoconference or/and clinic session	Weekly 60‐90-minute sessions (15‐30-minute caregiver/45‐60-minute youth); 2‐5 sessions	Facilitate trauma processing with caregiver support and compassionate engagement
Integration and recovery			
(C7) In vivo mastery with compassion	Videoconference or/and clinic session	Weekly 60-minute sessions; 1‐4 sessions	Promote real-life exposure with compassion to reduce avoidance and increase agency
(C8) Conjoint child-parent session	Clinic session	One 60‐90-minute joint session	Strengthen relational safety, mutual compassion, and emotional connectedness
(C9) Future safety and compassionate imagination	Videoconference or/and clinic session	One 60‐90-minute final session	Support future orientation, relapse prevention, and valued direction through compassion

a bTF-CBT-C: blended trauma-focused cognitive behavioral therapy with compassion. bTF-CBT-C is a flexible intervention that can be adapted to individual needs.

### Therapists and Implementation

All treatment will be delivered by licensed clinicians (psychologists or licensed psychotherapists) who have completed formal training in TF-CBT. Before initiating the study, therapists will complete a protocol-specific training program covering the structure of the blended intervention, digital delivery procedures, and the integration of compassion-focused strategies.

To ensure high-quality implementation and treatment fidelity, therapists will receive regular supervision from senior TF-CBT and CFT clinicians throughout the trial. Therapists will also receive brief guidance on culturally responsive and context-sensitive practice to ensure that the intervention is delivered consistently and safely across participating sites.

### Outcome Measures

#### Overview

Outcomes are prioritized in line with recommendations for pilot and feasibility trials. The primary focus is on feasibility and acceptability, while clinical and process-related outcomes are exploratory and intended to inform estimates of variability and guide outcome selection for a future trial. All outcome assessments will follow the predefined SPIRIT schedule (Table 1), with weekly and mid-module measures described in Multimedia Appendix 1. To minimize participant burden, exploratory measures are collected at selected assessment time points.

As some instruments (eg, Trauma-Related Shame Inventory and Trauma-Related Guilt Inventory) have not yet been formally validated in Swedish samples, their performance will be explored within the pilot data. This will include examination of distributions, internal consistency (eg, Cronbach α), and patterns of missing data to assess feasibility and interpretability. Sensitivity analyses will also be conducted to explore the robustness of findings across measures, and results will be interpreted with appropriate caution.

#### Primary Outcomes

##### Feasibility

###### Overview

Feasibility will be evaluated by assessing the extent to which the intervention and study procedures can be delivered within routine child and adolescent psychiatric services. Indicators will include recruitment rate, retention at each major assessment point, adherence to therapist-led and digital components, data completeness, and the functioning of study logistics such as randomization and assessment procedures. Adverse events will be monitored throughout the study via youth, caregiver, and therapist reports.

###### Adverse Event Monitoring

Adverse events related to digital and therapeutic components will be monitored using brief weekly screening items completed by youth, caregivers, and therapists. Respondents will report occurrence and rate severity on a 0‐3 scale. Events will be documented and reviewed in accordance with safety procedures.

### Acceptability

#### Overview

Acceptability will be assessed using multiple indicators capturing participants’ and caregivers’ perspectives on the intervention and study procedures. Measures will include treatment satisfaction, therapeutic alliance (youth, caregiver, and therapist versions), ratings of digital treatment usability and engagement, and evaluation of individual exercises. In addition, qualitative interviews with adolescents, caregivers, and therapists will explore perceived helpfulness, burden, and acceptability of the blended format.

#### Therapeutic Alliance

Therapeutic alliance will be measured using a brief 6-item questionnaire based on Bordin’s working alliance theory and adapted from versions of the Working Alliance Inventory [44]. Items will assess agreement on goals, collaboration on tasks, and the emotional bond using a 7-point scale (0‐6), where higher scores indicate a stronger alliance. Youth, caregivers, and therapists will complete parallel forms. Although the adapted version has not undergone formal validation, its use is considered appropriate for a pilot feasibility study, as it provides a brief, pragmatic assessment of alliance across respondent groups, consistent with recommendations for pilot and feasibility trials [34].

#### Digital Treatment Evaluation

Digital treatment evaluation will assess usability, engagement, and perceived helpfulness of the web-based components. Youth and caregivers will complete an 11-item scale rated on a 5-point Likert scale (0‐4), informed by established usability and technology acceptance frameworks. Higher scores will indicate greater usability, satisfaction, and perceived therapeutic value.

#### Exercise Evaluation

The exercise evaluation will capture youths’ and caregivers’ experiences with the exercises introduced during the web-based stabilization phase. Each exercise will be rated from −2 (very negative) to +2 (very positive), with “X” indicating noncompletion. The measure will provide insight into acceptability and identify exercises requiring refinement. Higher scores indicate a more positive experience, while consistently negative ratings may signal the need for modification.

#### Treatment Satisfaction (Youth)

Treatment satisfaction will be assessed using a structured measure that evaluates perceived helpfulness, quality of the therapeutic relationship, satisfaction with delivery formats (web-based application, video, and in-person), and perceived improvement. Items will be rated on Likert-type scales, with optional free-text responses. Higher scores will represent greater satisfaction.

### Exploratory and Clinical Outcomes

#### Overview

Exploratory clinical outcomes will be collected to examine preliminary patterns of symptom change, estimate variability, and inform outcome selection and sample-size calculations for a future fully powered noninferiority trial. Consistent with recommendations for pilot and feasibility studies, these analyses will be descriptive in nature and will not include formal hypothesis testing.

#### Child and Adolescent Trauma Screen, Version 2

The Child and Adolescent Trauma Screen, version 2 (CATS-2), will measure trauma exposure and PTSD symptoms. The instrument includes a checklist of 16 potentially traumatic events and 25 symptom items assessing re-experiencing, avoidance, negative mood/cognition, and hyperarousal. Symptoms are rated on a 4-point scale (0‐3), with total scores ranging from 0 to 60. CATS-2 has demonstrated strong reliability and validity in child and adolescent populations [45].

#### Children’s Revised Impact of Event Scale, 13-Item Version

The Children’s Revised Impact of Event Scale, 13-item version (CRIES-13), will assess posttraumatic stress reactions, including intrusion, avoidance, and arousal. The scale contains 13 items rated on a 4-point scale (0, 1, 3, and 5), yielding a total score of 0‐65, with higher scores indicating greater symptom severity. CRIES-13 has demonstrated good reliability and validity across youth samples, including in Swedish clinical contexts [46].

#### Dissociation Screening Questionnaire, 12-Item Version

The Dissociation Screening Questionnaire, 12-item version (DSQ-12), will screen for psychoform and somatoform dissociative symptoms. It consists of 12 items rated on a 5-point Likert scale (0‐4), with higher scores indicating more severe dissociation. DSQ-12 was developed and validated in Swedish adolescent samples and demonstrates strong reliability, convergent validity, and a stable factor structure [47].

#### Compassionate Engagement and Action Scales for Youth – Swedish Version

The Compassionate Engagement and Action Scales for Youth – Swedish Version (CEASY-SE) will measure compassion-related processes across 3 flows: compassion for self, compassion for others, and compassion received from others. Items are scored on a 10-point scale (1‐10), with higher scores reflecting stronger compassion skills. The Swedish adolescent version has shown good internal consistency and construct validity in both community and clinical samples [25,26].

#### Difficulties in Emotion Regulation Scale, 16-Item Version

The Difficulties in Emotion Regulation Scale, 16-item version (DERS-16), will assess broad difficulties in emotion regulation across 6 domains, including nonacceptance of emotions, limited access to strategies, and difficulty engaging in goal-directed behavior when distressed. The 16 items are rated on a 5-point scale (1-5), with higher scores reflecting greater dysregulation. DERS-16 has demonstrated strong reliability and validity across adolescent and clinical populations [48].

#### Montgomery-Åsberg Depression Rating Scale for Youth

The Montgomery-Åsberg Depression Rating Scale for Youth (MADRS-Y) will assess depressive symptoms and suicidality through 9 items scored on a 7-point scale (0‐6), yielding total scores from 0 to 54. Items assess sadness, concentration, lassitude, and sleep disturbances, with one item specifically targeting suicidal thoughts. The Swedish validation demonstrates good reliability and clinical utility in adolescent psychiatric samples [49].

#### Revised Child Anxiety and Depression Scale, 47-Item Version

The Revised Child Anxiety and Depression Scale, 47-item version (RCADS-47), will measure symptoms across 6 domains: separation anxiety, social phobia, generalized anxiety, panic disorder, obsessive-compulsive symptoms, and major depression. Items are rated on a 4-point scale (0‐3), with higher scores indicating greater severity. RCADS has strong psychometric support internationally and has been widely used in Nordic youth contexts [50].

#### Trauma-Related Shame Inventory

The Trauma-Related Shame Inventory (TRSI) will assess shame-based cognitive and emotional responses to trauma, including self-blame, negative self-evaluation, feelings of exposure, and avoidance urges. Items are rated on a 5-point scale (0‐4). TRSI has demonstrated adequate reliability and validity among trauma-exposed youth and adults, although no Swedish validation is currently available [16].

#### Trauma-Related Guilt Inventory

The Trauma-Related Guilt Inventory (TRGI) will measure guilt cognitions, emotional distress, and avoidance behaviors following traumatic events. The 32 items are rated on a 5-point Likert scale (0‐4), producing both subscale and total scores. TRGI has shown strong psychometric properties across diverse trauma-exposed populations, though no Swedish validation exists [51].

#### Parental Stress Scale

The Parental Stress Scale will assess caregivers’ emotional stress related to the parenting role, including perceived rewards, demands, and strain. The scale contains 18 items rated on a 5-point scale (1-5), with higher scores indicating greater parental stress. A recent Swedish validation demonstrates good internal consistency and a robust 2-factor structure in both community and clinical samples [52].

### Analysis

#### Quantitative Analysis

All quantitative analyses will follow the intention-to-treat principle [53], including all randomized participants in their assigned condition. Consistent with recommendations for pilot and feasibility trials, analyses will focus on estimating feasibility and acceptability metrics using descriptive statistics (eg, proportions, means, and 95% CIs). These outcomes will be interpreted in relation to predefined progression criteria to inform decisions about trial procedures.

Clinical and process outcomes will be analyzed descriptively, with a focus on estimation and patterns rather than formal hypothesis testing. To support interpretability, these outcomes will be organized into predefined conceptual domains (eg, clinical symptoms, compassion-related processes, and emotion regulation) and interpreted in terms of overall patterns across related measures rather than individual statistical significance.

Exploratory clinical outcomes will be examined to describe symptom trajectories and estimate variability, thereby informing the design of a future, fully powered noninferiority trial. Baseline-adjusted analysis of covariance models and linear mixed effects models will be used descriptively to examine patterns of change over time across study conditions and to generate variance estimates and confidence intervals, rather than to formally test between-group differences. Individual-level changes will be assessed using the Reliable Change Index [54,55]. All analyses will be conducted using standard statistical software (eg, R or SPSS).

#### Missing Data

Patterns of missing data will be examined to evaluate the assumptions of missing at random. For exploration analyses, multiple imputation will be applied as appropriate. Mixed effects models will use full-information maximum likelihood estimation, which accommodates incomplete data.

#### Process and Mechanism Measures

Weekly and mid-module process measures (eg, alliance, digital engagement, exercise evaluations, and mechanisms related to shame and guilt) will be summarized descriptively and explored in relation to symptom patterns.

#### Qualitative Analysis

Qualitative interviews with adolescents, caregivers, and therapists will be analyzed using reflexive thematic analysis following Braun and Clarke [56,57]. The analysis will primarily adopt an inductive, experiential-semantic approach, focusing on participants’ accounts of feasibility, acceptability, and engagement while allowing for interpretative depth in identifying patterns across participant groups. Reflexivity will be actively integrated, with researchers reflecting on how their clinical and theoretical perspectives may influence the analytic process [56,57]. Given the inclusion of adolescents, caregivers, and therapists, both within-group and cross-group patterns will be explored to identify shared and divergent perspectives.

### Progression Criteria

Progression to a fully powered noninferiority RCT will be determined based on predefined feasibility criteria established a priori, in accordance with CONSORT recommendations for pilot and feasibility studies. The pilot trial will be considered feasible to progress if the following thresholds are met: (1) recruitment: ≥60% of eligible participants enroll; (2) retention: ≥70% of enrolled participants complete the postintervention assessment; (3) acceptability: mean ratings of ≥4/5 from adolescents and caregivers on satisfaction and usability measures; and (4) data completeness: ≥80% of planned data points are collected across core assessment points.

These thresholds are consistent with feasibility patterns observed in comparable interventions. For example, blended trauma-focused cognitive therapy trials have reported high retention (approximately 88%) and strong satisfaction ratings (approximately 4.5/5), as in routine psychiatric care [58]. Similarly, digital PTSD interventions for youth have used recruitment, adherence, and retention as primary feasibility markers, with retention typically ranging from 70% to 90% [59]. Additional digital trauma-focused programs for adolescents and young adults report comparable feasibility patterns [12]. Together, these findings support the feasibility thresholds selected for the present pilot trial.

These criteria will guide decisions regarding whether the intervention and trial procedures are sufficiently feasible and acceptable to proceed to a fully powered trial. If one or more thresholds are not met, the research team will review barriers and determine whether procedural, methodological, or intervention-specific modifications are required before advancing to the next phase.

## Results

**Figure 1.**
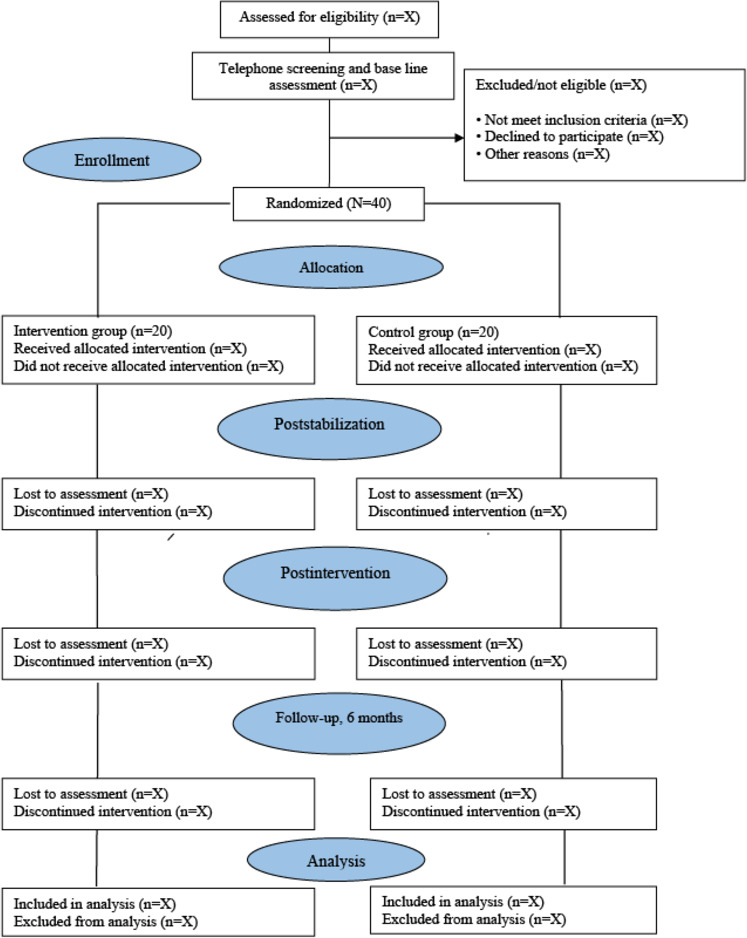
CONSORT flowchart of participants.

Recruitment for the pilot trial started in February 2026, with data collection expected to be completed by December 2028. Results will be reported in accordance with the CONSORT extension for pilot and feasibility trials, including a CONSORT flow diagram illustrating participant progression through the study (see Figure 1).

Feasibility and acceptability outcomes will be presented using descriptive statistics (eg, proportions, means, and 95% CIs) across assessment time points. Estimates of variance in clinical outcomes will be used to inform sample size calculations for a future fully powered noninferiority trial, including the specification of an appropriate noninferiority margin. Exploratory outcomes will be reported as patterns of change over time using descriptive summaries and graphical representations of symptom trajectories. Additional visualizations (eg, line plots of symptom change and summary figures of feasibility indicators) will be used to enhance interpretability.

Qualitative findings will be presented as themes developed through reflexive thematic analysis and integrated with quantitative indicators to provide a comprehensive understanding of feasibility, acceptability, and user experiences. Together, these results will inform the refinement of the intervention and the key design parameters for a future fully powered trial. If predefined feasibility thresholds are not met (eg, retention below prespecified levels), the research team will review potential barriers and consider modifications to study procedures or intervention delivery prior to a future trial.

## Discussion

This protocol presents a blended, compassion-informed adaptation of TF-CBT (bTF-CBT-C) for adolescents with PTSD and describes its planned evaluation in routine child and adolescent psychiatric services in northern Sweden. The intervention is designed to address key barriers to trauma-focused treatment in rural and sparsely populated regions, including limited access to specialized care and challenges related to sustained emotional engagement in trauma-focused work.

The pilot RCT will contribute in several ways. It will generate feasibility and acceptability data on delivering a blended trauma-focused intervention in routine services, where flexible delivery formats are important for promoting equity in care [12,58]. In addition, the systematic integration of compassion-focused strategies targeting mechanisms such as shame, self-criticism, and threat regulation may support engagement in trauma-focused work and address key barriers to participation in standard TF-CBT [20-22]. Importantly, these processes are transdiagnostic and implicated across a range of internalizing disorders, suggesting broader applicability beyond PTSD. The intervention has been developed with input from youth, caregivers, therapists, and user representatives, enhancing ecological validity [5,41]. The inclusion of weekly digital process measures further enables exploration of dynamic mechanisms and their relationship to treatment engagement [12,58].

Several methodological strengths enhance the study’s rigor. The trial follows established guidelines for pilot and feasibility studies and combines structured diagnostic assessment with a mixed methods design to triangulate quantitative and qualitative findings. The blended format is built on a CE-marked digital platform integrated into routine care, supported by therapist training and supervision to promote consistent delivery. Cultural and contextual adaptations, including attention to Sámi perspectives and local norms, aim to enhance the intervention’s relevance and acceptability in the study setting [7].

The study also has limitations inherent to pilot designs. The small sample size precludes conclusions regarding clinical effectiveness, and the trial is not powered to detect group differences. As in most psychological treatment trials, therapists and participants are not blinded to treatment allocation, which may introduce expectancy or performance-related biases. Qualitative interviews will explore participants’ and therapists’ experiences and expectations, providing contextual insight into these influences.

Variability in therapist delivery across regions may influence treatment fidelity. This will be addressed through standardized training delivered jointly across sites, structured supervision, and session logs. Although formal fidelity ratings are not included in this pilot study, documentation of treatment delivery and adaptations will provide insights into intervention consistency. This approach was chosen to minimize the burden on participants and therapists during this initial feasibility study.

Implementation in rural northern Sweden presents specific challenges. The use of a blended format introduces potential risks related to the digital divide, including variability in broadband access and digital literacy in remote areas. To mitigate these challenges, the intervention allows tailored delivery formats that combine web-based application, videoconferencing, and in-person sessions, supported by technical assistance from participating clinics. If barriers to digital participation are identified, delivery will be adjusted as needed (eg, increased use of in-person sessions or additional therapist support).

Although the study is conducted in a Swedish rural context, the challenges addressed, limited access to specialized trauma care and geographic dispersion, are shared across many settings globally. As such, the blended model may be relevant for scaling trauma-focused interventions in other rural and underserved settings. Ongoing qualitative data and user involvement will provide insight into context-specific barriers and inform iterative adaptations to optimize implementation in routine care.

Findings from the pilot trial will directly inform the design of a future noninferiority RCT. Feasibility and acceptability outcomes will guide refinements of recruitment strategies, intervention delivery, and therapist training, while estimates of variability will support sample size calculations and specification of an appropriate noninferiority margin. Qualitative findings will complement these data by identifying contextual and relational factors influencing engagement.

Taken together, this study points to the potential value of integrating compassion-focused, mechanism-targeted approaches within trauma-focused interventions to support engagement in treatment among adolescents. The pilot trial will provide critical feasibility data to inform the design of a future non-inferiority randomized controlled trial and represents an important step toward developing scalable, evidence-based trauma-focused interventions with the potential to advance health equity in underserved adolescent populations.

## Supplementary material

10.2196/92270 Multimedia Appendix 1 Detailed assessment schedule.

10.2196/92270 Multimedia Appendix 2 Screenshots from the adolescent version of the app showing (left to right) psychoeducation about self-critical thinking and the threat system, a guided compassion exercise (“SNÄLLA – six steps of self-compassion”), and a reflective writing task (in Swedish).

10.2196/92270 Checklist 1 SPIRIT checklist.

## Abbreviations

bTF-CBT-C: blended trauma-focused cognitive behavioral therapy with compassion
CEASY-SE: Compassionate Engagement and Action Scales for Youth – Swedish Version
CFT: compassion-focused therapy
CONSORT: Consolidated Standards of Reporting Trials
CRIES-13: Children’s Revised Impact of Event Scale, 13-item version
*DSM-5*: *Diagnostic and Statistical Manual of Mental Disorders* (Fifth Edition)
DSQ-12: Dissociation Screening Questionnaire, 12-item version
FAIR: findable, accessible, interoperable, and reusable principles
GDPR: General Data Protection Regulation
MADRS-Y: Montgomery-Åsberg Depression Rating Scale for Youth
MINI-KID: Mini-International Neuropsychiatric Interview for Children and Adolescents
MOST: multiphase optimization strategy
PRACTICE: psychoeducation, relaxation, affective modulation, cognitive coping, trauma narrative/exposure, in vivo mastery, conjoint caregiver-child sessions, and enhancing safety and future development components
PTSD: posttraumatic stress disorder
RCADS-47: Revised Child Anxiety and Depression Scale, 47-item version
RCT: randomized controlled trial
SPIRIT: Standard Protocol Items: Recommendations for Interventional Trials
TF-CBT: trauma-focused cognitive behavioral therapy
TRGI: Trauma-Related Guilt Inventory
TRSI: Trauma-Related Shame Inventory

## References

[1] Visser I, van der Mheen M, Dorsman H (2026). Post-traumatic stress disorder rates in trauma-exposed children and adolescents: updated three-level meta-analysis. Br J Psychiatry.

[2] Tamir TT, Tekeba B, Mekonen EG, Gebrehana DA, Zegeye AF (2025). Shadows of trauma: an umbrella review of the prevalence and risk factors of post-traumatic stress disorder in children and adolescents. Child Adolesc Psychiatry Ment Health.

[3] (2024). Waiting times in healthcare. https://extra.skr.se/vantetiderivarden/vantetidsstatistik.63530.html.

[4] Golestani R, Farahani FK, Peters P (2025). Exploring barriers to accessing health care services by young women in rural settings: a qualitative study in Australia, Canada, and Sweden. BMC Public Health.

[5] Wallin L, Lundqvist U, Svedin CG, Dennhag I (2024). “Longing to be cared about and cared for” Exploring experiences of trauma therapy and views on future trauma therapy (including digital) for young people in rural northern Sweden. Child Youth Serv Rev.

[6] Jonsson F, Christianson M, Wiklund M, Hurtig AK, Goicolea I (2021). Collective imaginaries of caring landscapes for rural youth: a concept mapping study in northern Sweden. BMC Public Health.

[7] Goicolea I, Gotfredsen AC, Jonsson F, Wernesjö U (2023). The promise of belonging: racialized youth subject positions in the Swedish rural north. J Int Migr Integr.

[8] Jonsson F, Goicolea I, Christianson M, Carson DB, Wiklund M (2020). Landscapes of care and despair for rural youth – a qualitative study in the northern Swedish “periphery”. Int J Equity Health.

[9] Dworkin ER (2020). Risk for mental disorders associated with sexual assault: a meta-analysis. Trauma Violence Abuse.

[10] Cohen JA, Mannarino AP (2022). Trauma-focused cognitive behavioral therapy for children and families. Child Adolesc Psychiatr Clin N Am.

[11] Hoppen TH, Wessarges L, Jehn M (2025). Psychological interventions for pediatric posttraumatic stress disorder: a systematic review and network meta-analysis. JAMA Psychiatry.

[12] Schulte C, Harrer M, Sachser C, Weiss J, Zarski AC (2024). Internet- and mobile-based psychological interventions for post-traumatic stress symptoms in youth: a systematic review and meta-analysis. NPJ Digit Med.

[13] Rajan G, Ljunggren G, Wändell P, Wahlström L, Svedin CG, Carlsson AC (2020). Health care consumption among adolescent girls prior to diagnoses of sexual abuse, a case-control study in the Stockholm Region. Eur Child Adolesc Psychiatry.

[14] Brinckman B, Alfaro E, Wooten W, Herringa R (2024). The promise of compassion-based therapy as a novel intervention for adolescent PTSD. J Affect Disord Rep.

[15] Potts C, Kealy C, McNulty JM (2025). Digital mental health interventions for young people aged 16-25 years: scoping review. J Med Internet Res.

[16] Øktedalen T, Hoffart A, Langkaas TF (2015). Trauma-related shame and guilt as time-varying predictors of posttraumatic stress disorder symptoms during imagery exposure and imagery rescripting – a randomized controlled trial. Psychother Res.

[17] Blakemore SJ, Mills KL (2014). Is adolescence a sensitive period for sociocultural processing?. Annu Rev Psychol.

[18] Dahl RE (2016). The developmental neuroscience of adolescence: revisiting, refining, and extending seminal models. Dev Cogn Neurosci.

[19] Dahl RE, Allen NB, Wilbrecht L, Suleiman AB (2018). Importance of investing in adolescence from a developmental science perspective. Nature.

[20] Gilbert P (2014). The origins and nature of compassion focused therapy. Br J Clin Psychol.

[21] Petrocchi N, Ottaviani C, Cheli S (2023). The impact of compassion-focused therapy on positive and negative mental health outcomes: results of a series of meta-analyses. Clin Psychol Sci Pract.

[22] Vestin M, Wallin L, Naesström M (2025). Internet-based group compassion-focused therapy for Swedish young people with stress, anxiety and depression: a pilot waitlist randomized controlled trial. Front Psychol.

[22] Vestin M, Jokinen J, Blomqvist I, Dennhag I (2025). Participants’ evaluation of an internet-based group compassion-focused therapy program for young people in Sweden. Front Psychol.

[24] Egan SJ, Rees CS, Delalande J (2022). A review of self-compassion as an active ingredient in the prevention and treatment of anxiety and depression in young people. Adm Policy Ment Health.

[25] Henje E, Rindestig FC, Gilbert P, Dennhag I (2020). Psychometric validity of the Compassionate Engagement and Action Scale for Adolescents: a Swedish version. Scand J Child Adolesc Psychiatr Psychol.

[26] Wallin L, Svedin CG, Wiberg M, Dennhag I (2025). The Compassionate Engagement and Action Scale for Youths: psychometric properties in a clinical psychiatric Swedish sample. Front Psychol.

[27] Axelsson E (2025). Equivalence and non-inferiority trials in the evaluation of non-pharmacological interventions: rationale, challenges and recommendations. BMJ Open.

[28] Chan AW, Boutron I, Hopewell S (2025). SPIRIT 2025 statement: updated guideline for protocols of randomised trials. PLoS Med.

[29] Thabane L, Lancaster G (2019). A guide to the reporting of protocols of pilot and feasibility trials. Pilot Feasibility Stud.

[30] Stewart RW, Orengo-Aguayo RE, Cohen JA, Mannarino AP, de Arellano MA (2017). A pilot study of trauma-focused cognitive-behavioral therapy delivered via telehealth technology. Child Maltreat.

[31] Vigerland S, Lenhard F, Bonnert M (2016). Internet-delivered cognitive behavior therapy for children and adolescents: a systematic review and meta-analysis. Clin Psychol Rev.

[32] Barton GH (2020). Designing and Conducting Mixed Methods Research (3rd Edition; International Student Edition) by John W. Creswell & Vicki L. Plano Clark [Book review]. Cogn Psychol Bull.

[33] Fetters MD, Curry LA, Creswell JW (2013). Achieving integration in mixed methods designs – principles and practices. Health Serv Res.

[34] Eldridge SM, Chan CL, Campbell MJ (2016). CONSORT 2010 statement: extension to randomised pilot and feasibility trials. BMJ.

[35] Ying X, Freedland KE, Powell LH, Stuart EA, Ehrhardt S, Mayo-Wilson E (2025). Determining sample size for pilot trials: a tutorial. BMJ.

[36] Whitehead AL, Julious SA, Cooper CL, Campbell MJ (2016). Estimating the sample size for a pilot randomised trial to minimise the overall trial sample size for the external pilot and main trial for a continuous outcome variable. Stat Methods Med Res.

[37] Lancaster GA, Dodd S, Williamson PR (2004). Design and analysis of pilot studies: recommendations for good practice. J Eval Clin Pract.

[38] Amni Care. Stretch Care.

[39] Goldstein CM, Kugler KC, Gellman MD (2019). Encyclopedia of Behavioral Medicine.

[40] Collins LM (2018). Optimization of Behavioral, Biobehavioral, and Biomedical Interventions: The Multiphase Optimization Strategy (MOST).

[41] Om Hjärnkoll. Hjärnkoll.

[42] Cohen JA, Mannarino AP (2015). Trauma-focused cognitive behavior therapy for traumatized children and families. Child Adolesc Psychiatr Clin N Am.

[43] Cohen J, Mannarino A, Deblinger E (2017). Treating Trauma and Traumatic Grief in Children and Adolescents.

[44] Horvath AO, Greenberg LS (1989). Development and validation of the Working Alliance Inventory. J Couns Psychol.

[45] Sachser C, Berliner L, Risch E (2022). The Child and Adolescent Trauma Screen 2 (CATS-2) – validation of an instrument to measure DSM-5 and ICD-11 PTSD and complex PTSD in children and adolescents. Eur J Psychotraumatol.

[46] Perrin S, Meiser-Stedman R, Smith P (2005). The Children’s Revised Impact of Event Scale (CRIES): validity as a screening instrument for PTSD. Behav Cogn Psychother.

[47] Nilsson D, Green S, Svedin CG, Dahlström Ö (2019). Psychoform and somatoform dissociation among children and adolescents: an evaluation of a new short screening instrument for dissociation, DSQ-12. Eur J Trauma Dissociation.

[48] Bjureberg J, Ljótsson B, Tull MT (2016). Development and validation of a brief version of the Difficulties in Emotion Regulation Scale: the DERS-16. J Psychopathol Behav Assess.

[49] Vestin M, Blomqvist I, Henje E, Dennhag I (2024). Psychometric validation of the Montgomery-Åsberg Depression Rating Scale – Youth (MADRS-Y) in a clinical sample. Nord J Psychiatry.

[50] Esbjørn BH, Sømhovd MJ, Turnstedt C, Reinholdt-Dunne ML (2012). Assessing the Revised Child Anxiety and Depression Scale (RCADS) in a national sample of Danish youth aged 8-16 years. PLoS One.

[51] Kubany ES, Haynes SN, Abueg FR, Manke FP, Brennan JM, Stahura C (1996). Development and validation of the Trauma-Related Guilt Inventory (TRGI). Psychol Assess.

[52] Lindström T, Bergman TH, Annerstedt M, Forster M, Bölte S, Hirvikoski T (2024). Psychometric properties of the Parental Stress Scale in Swedish parents of children with and without neurodevelopmental conditions. Scand J Child Adolesc Psychiatr Psychol.

[53] Gupta SK (2011). Intention-to-treat concept: a review. Perspect Clin Res.

[54] Jacobson NS, Truax P (1991). Clinical significance: a statistical approach to defining meaningful change in psychotherapy research. J Consult Clin Psychol.

[55] Blampied NM (2022). Reliable change and the reliable change index: still useful after all these years?. Cogn Behav Ther.

[56] Braun V, Clarke V (2022). Thematic Analysis: A Practical Guide.

[57] Braun V, Clarke V (2019). Reflecting on reflexive thematic analysis. Qual Res Sport Exerc Health.

[58] Lundin J, Jansson-Fröjmark M, Gustafsson-Björverud L (2024). Integrating digital and in-person therapy for PTSD: feasibility and acceptability of blended trauma-focused cognitive therapy in routine care. Front Psychiatry.

[59] Smith P, Ehlers A, Carr E (2022). Therapist-supported online cognitive therapy for post-traumatic stress disorder (PTSD) in young people: protocol for an early-stage, parallel-group, randomised controlled study (OPTYC trial). BMJ Open.

