# Supplementary material for: Blended Trauma-Focused Cognitive Behavioral Therapy With Compassion for Adolescents With Posttraumatic Stress Disorder: Protocol for a Pilot Randomized Controlled Trial in Northern Sweden
Source: JMIR Res Protoc. 2026 Jul 15;15:e92270. doi: 10.2196/92270 (PMC13372296; doi:10.2196/92270)
Supplement: Multimedia Appendix 1 [file resprot-v15-e92270-s001.pdf]

			Stabilization Phase C0-C5		Post-Stabilization C6-C9		Post-Intervention	
	Enrollment	Allocation	Week 1 + one session*	Week 2, 3, 4, 5	Week 5-7 Completed stabilization+ one session*	Week 10-12	Completed intervention	6-month follow-up
**Timepoint**	T0		T1	T2-T5	T6	T7	T8	T9
Eligibility screen	X							
Informed consent	X							
Randomization		X						
**INTERVENTIONS**								
bTF-CBT-C		X	X	X	X	X	X	X
Standard TF-CBT		X	X	X	X	X	X	X
**PRIMARY ASSESSMENT**								
**Feasibility:**			X	X	X	X	X	
Recruitment/retention	X	X			X		X	
Adherence			X	X	X	X	X	
Adverse Event			X	X	X	X	X	
**Acceptability:**								
Therapeutic alliance			X		X	X	X	
Digital Evaluation			X	X				
Exercise Evaluation			X	X				
Treatment Satisfaction							X	
Qualitative interviews					X		X	X
**EXPLORATORY & CLINICAL OUTCOMES**								
Demographic questions	X							
PTSD:								
CATS-2	X				X		X	X
CRIES	X		X		X		X	
Dissociation: DSQ-12	X						X	X
Compassion: CEASY-SE	X		X		X		X	X
Depression/ Anxiety:								
RCADS	X				X		X	X
MADRS-Y (Suicide)	X				X		X	X
Parental stress	X				X		X	X
Emotion regulation (DERS-16)	X				X		X	X

Shame:								
VAS-scale	X	X		X	X		X	X
TRSI/TRGI	X				X		X	X

Note. *Video conference or clinic-based session.
Appendix A provides detailed assessment time points beyond the core SPIRIT timepoints (T0–T3).
Therapist adherence will be monitored through session recordings and adherence checklists.
CATS-2 = Child and Adolescent Trauma Screen; CEASY-SE = Compassionate Engagement and Action Scales – Swedish version; CRIES = Children's Revised Impact of Event Scale; DERS = Difficulties in Emotion Regulation Scale; DSQ-12 = Dissociative Symptoms Questionnaire; MADRS-Y = Montgomery-Åsberg Depression Rating Scale for Youth; PSS = Parental Stress Scale; RCADS = Revised Child Anxiety and Depression Scale; TRSI = Trauma-Related Shame Inventory; TRGI = Trauma-Related Guilt Inventory; VAS = Visual Analogue Scale
