# Supplementary figures and images for: Blended Trauma-Focused Cognitive Behavioral Therapy With Compassion for Adolescents With Posttraumatic Stress Disorder: Protocol for a Pilot Randomized Controlled Trial in Northern Sweden
Source: JMIR Res Protoc. 2026 Jul 15;15:e92270. doi: 10.2196/92270 (PMC13372296; doi:10.2196/92270)

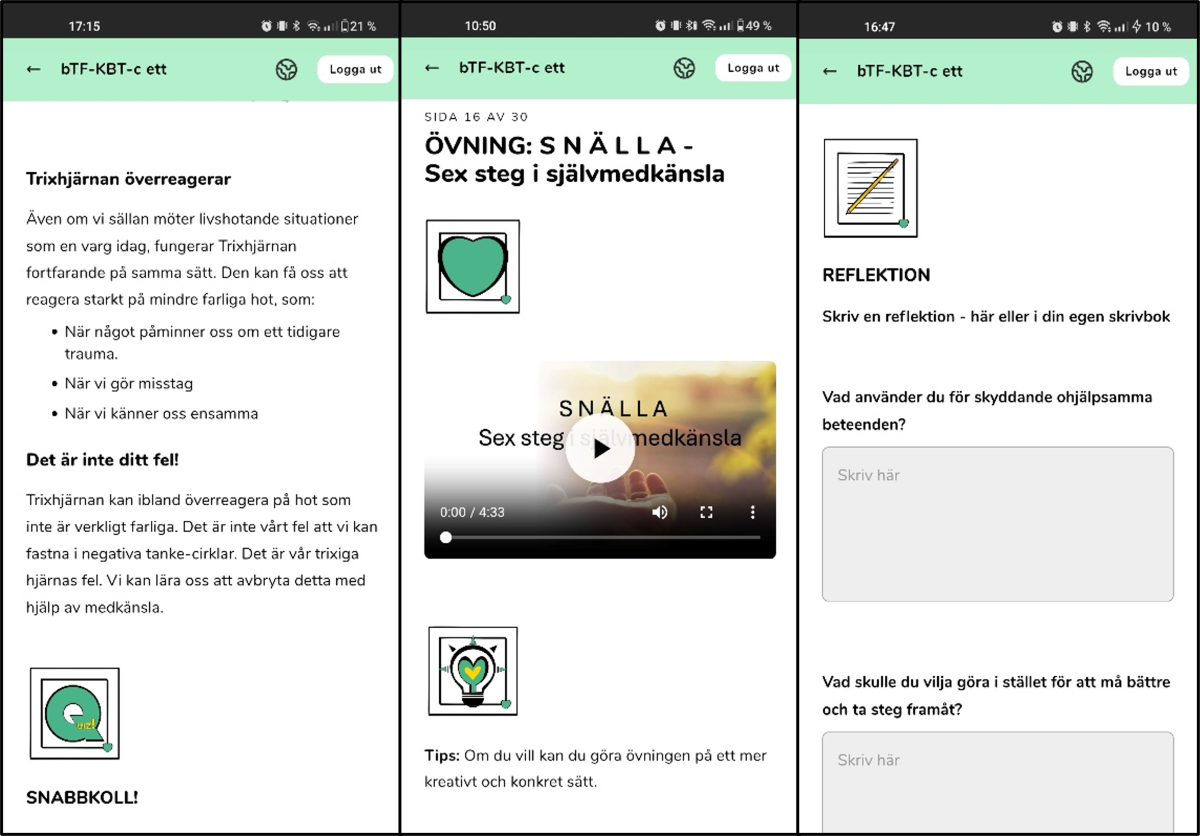

Supplement: Multimedia Appendix 2 [file resprot-v15-e92270-s002.png]
